# Supplementary material for: Characterization of Beeswax and Rice Bran Wax Oleogels Based on Different Types of Vegetable Oils and Their Impact on Wheat Flour Dough Technological Behavior during Bun Making
Source: Gels. 2024 Mar 12;10(3):194. doi: 10.3390/gels10030194 (PMC10970211; doi:10.3390/gels10030194)
Supplement: Supplementary file 1 [file gels-10-00194-s001.zip › gels-2875749-supplementary.pdf]

Supplementary Materials

Table S1A. DSC parameters of oleogel samples, oils and waxes – second heating profile.

Sample	Second heating, -60 to 100 °C , melting				Second heating, -60 to 100 °C , melting 2			
	Onset, °C	Midpoint, °C	End, °C	Enthalpy, J/g	Onset, °C	Midpoint, °C	End, °C	Enthalpy, J/g
GO_5BW	-32.83	-28.81	-13.93	25.88	40.36	49.37	58.53	1.72
GO_9BW	-32.75	-29.02	-14.92	26.87	42.85	51.90	61.43	2.96
HO_5BW	-37.49	-32.40	-27.61	7.08	40.97	48.99	58.05	1.22
HO_9BW	-37.94	-32.69	-27.92	7.74	40.73	51.56	61.49	2.99
OL_5BW	-16.69	-5.21	6.31	52.45	41.10	48.41	57.19	1.86
OL_9BW	-17.01	-5.71	4.10	62.05	41.32	51.52	59.55	3.30
SO_5BW	-31.94	-26.95	-12.05	34.91	38.71	49.55	58.88	2.49
SO_9BW	-32.21	-27.35	-11.40	36.11	42.03	51.71	60.48	3.84
WO_5BW	-49.28	-34.22	-24.05	32.06	39.29	49.34	56.86	1.75
WO_9BW	-47.28	-34.33	-23.95	32.92	41.45	51.93	62.36	3.31
GO_5RW	-32.21	-28.62	-16.10	21.97	56.13	65.86	75.16	5.13
GO_9RW	-32.42	-28.64	-14.93	22.91	55.21	67.79	73.64	8.84
HO_5RW	-37.71	-32.88	-28.16	10.22	55.31	67.32	75.28	5.65
HO_9RW	-38.37	-32.90	-27.92	11.72	53.77	69.93	76.23	13.47
OL_5RW	-16.49	-5.43	7.01	57.15	50.46	66.78	73.84	6.10
OL_9RW	-16.24	-5.26	5.50	65.45	48.96	66.47	73.63	10.58
SO_5RW	-31.32	-22.89	-7.47	35.32	51.88	66.06	72.06	5.43
SO_9RW	-31.04	-22.97	-7.65	40.77	52.02	67.32	72.58	11.63
WO_5RW	-51.86	-33.55	-23.96	35.28	53.90	62.96	68.85	3.20
WO_9RW	-52.42	-34.04	-25.05	36.58	52.96	68.97	74.70	14.05
GO	-32.16	-28.21	-12.58	29.82	-	-	-	-
HO	-37.27	-32.43	-20.76	17.87	-	-	-	-
OL	-27.03	-5.85	5.62	74.86	-	-	-	-
SO	-42.48	-25.96	-7.23	57.99	-	-	-	-
WO	-50.78	-34.14	-18.00	44.67	-	-	-	-
BW	-	-	-	-	33.72	62.43	68.61	165.33
RW	-	-	-	-	66.72	78.74	85.04	117.54

Table S1B. DSC parameters of oleogel samples, oils and waxes – second cooling profile.

Sample	Second cooling, 100 to 20 °C, phase transition					Second cooling, 100 to 20 °C, crystallization			
	Onset, °C	Midpoint, °C	End, °C	ΔCp, J/(g. °C)	Onset, °C	Onset, °C	Midpoint, °C	End, °C	Enthalpy, J/g
GO_5BW	47.50	44.71	41.60	0.0040	0.56	-	-	-	-
GO_9BW	45.23	48.27	52.50	0.0060	1.18	-	-	-	-
HO_5BW	48.49	44.72	41.37	0.0090	0.50	-	-	-	-
HO_9BW	54.36	48.19	45.37	0.0140	1.89	-	-	-	-
OL_5BW	47.43	44.43	42.39	0.0050	0.48	-	-	-	-
OL_9BW	51.17	47.48	44.94	0.0060	1.15	-	-	-	-
SO_5BW	51.02	45.59	41.81	0.0030	0.88	-	-	-	-
SO_9BW	53.82	48.79	46.00	0.0090	1.49	-	-	-	-
WO_5BW	48.71	44.51	41.66	0.0070	0.78	-	-	-	-
WO_9BW	54.05	48.69	45.46	0.0130	1.53	-	-	-	-
GO_5RW	67.59	61.84	57.80	0.0040	1.79	-	-	-	-
GO_9RW	72.30	63.70	61.90	0.0200	7.75	-	-	-	-
HO_5RW	70.34	64.37	61.34	0.0080	3.14	-	-	-	-
HO_9RW	75.82	67.65	65.41	0.0160	6.91	-	-	-	-
OL_5RW	66.21	62.26	60.17	0.0010	1.29	-	-	-	-
OL_9RW	73.14	61.62	60.13	0.0460	11.44	-	-	-	-
SO_5RW	69.17	61.93	59.02	0.0100	2.34	-	-	-	-
SO_9RW	72.20	64.56	61.55	0.0110	6.15	-	-	-	-
WO_5RW	58.98	53.45	51.51	0.0080	0.97	-	-	-	-
WO_9RW	76.19	65.86	64.11	0.0250	12.79	-	-	-	-
GO	-	-	-	-	-	-	-	-	-
HO	-	-	-	-	-	-	-	-	-
OL	-	-	-	-	-	-	-	-	-
SO	-	-	-	-	-	-	-	-	-
WO	-	-	-	-	-	-	-	-	-
BW	-	-	-	-	-	-	-	-	-
RW	84.51	75.61	73.98	0.0120	66.25	60.32	56.48	45.55	21.04

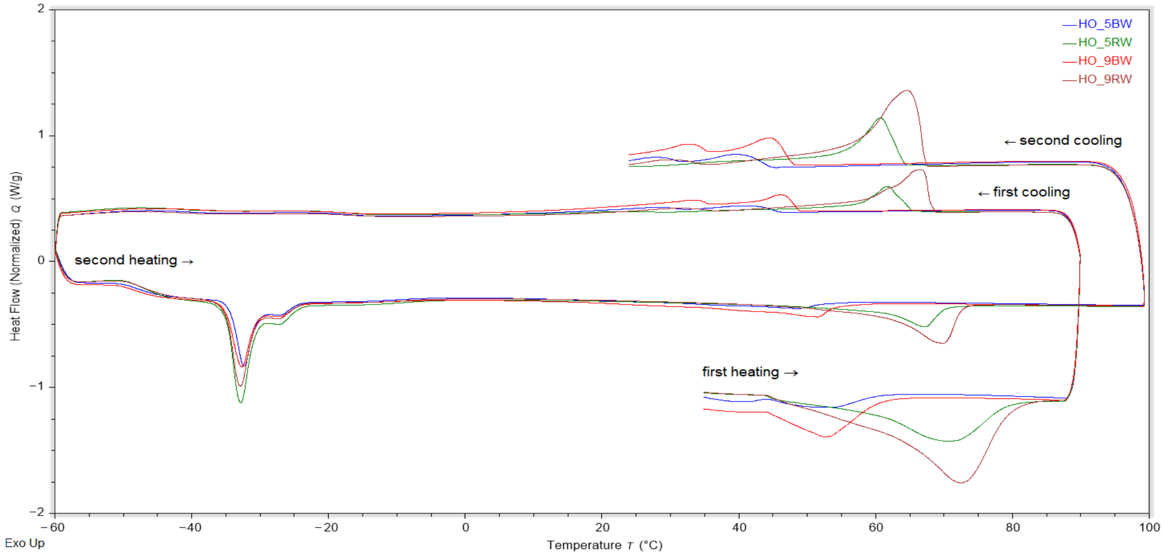
HO_5BW
HO_5RW
HO_9BW
HO_9RW
← second cooling
← first cooling
second heating →
first heating →
Heat Flow (Normalized) Q (W/g)
Temperature T (°C)
Exo Up

A

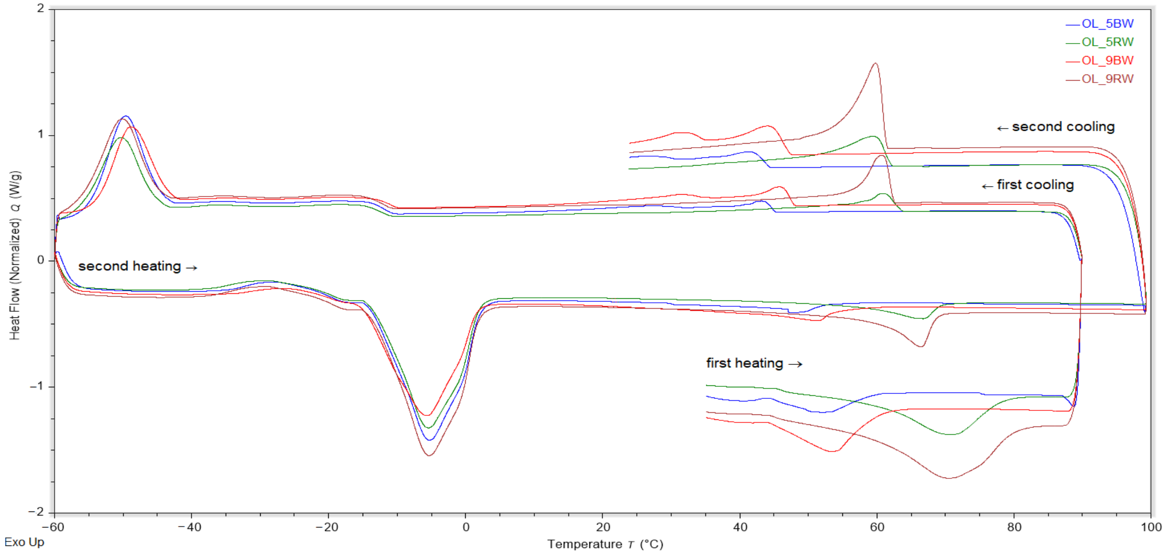
OL_5BW
OL_5RW
OL_9BW
OL_9RW
← second cooling
← first cooling
second heating →
first heating →
Heat Flow (Normalized) Q (W/g)
Temperature T (°C)
Exo Up

B

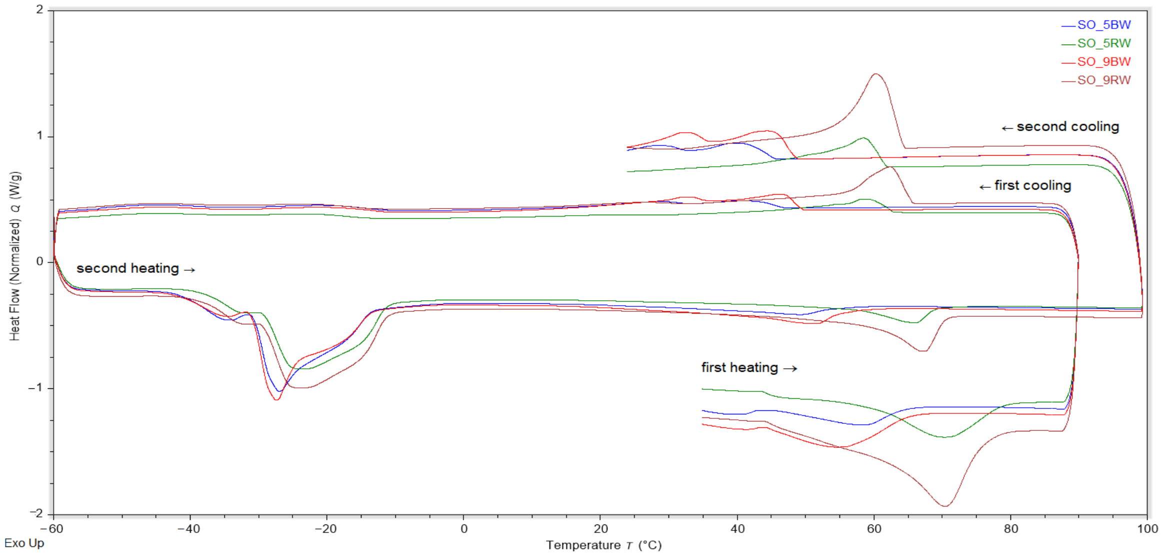

C

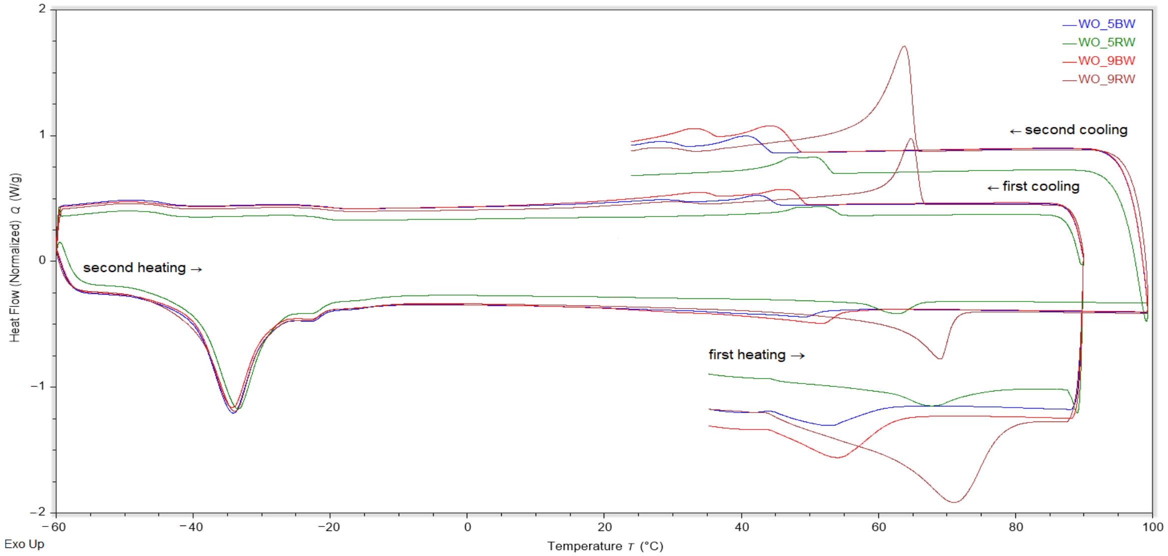

D

Figure S1. Thermograms of oleogels, oils, and waxes for heating (→) and cooling (←) profiles.

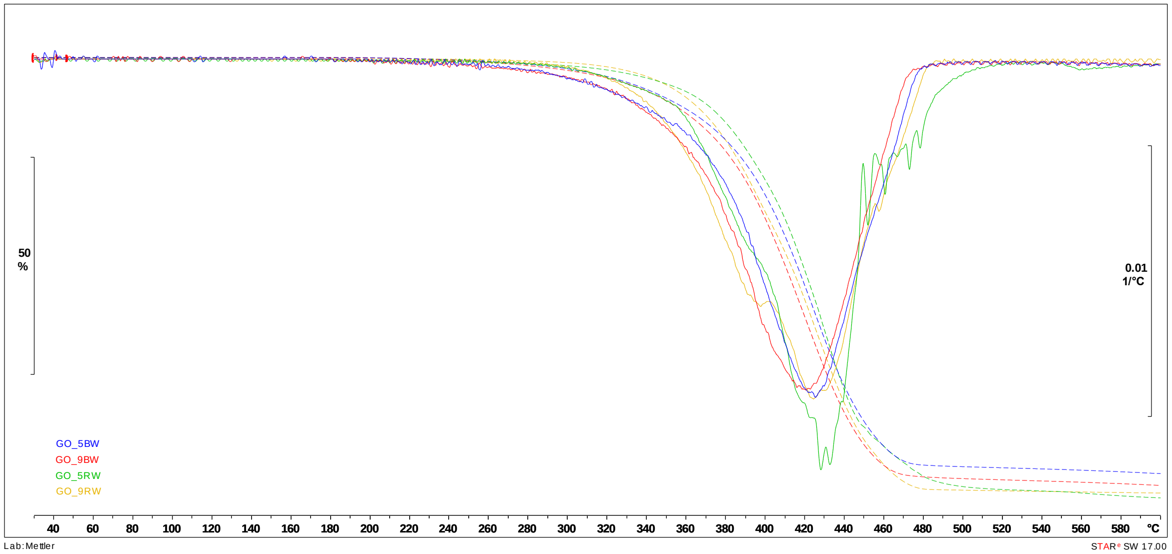

50
%
0.01
1/°C
GO_5BW
GO_9BW
GO_5RW
GO_9RW
40 60 80 100 120 140 160 180 200 220 240 260 280 300 320 340 360 380 400 420 440 460 480 500 520 540 560 580 °C
Lab:Mettler
STAR® SW 17.00

A

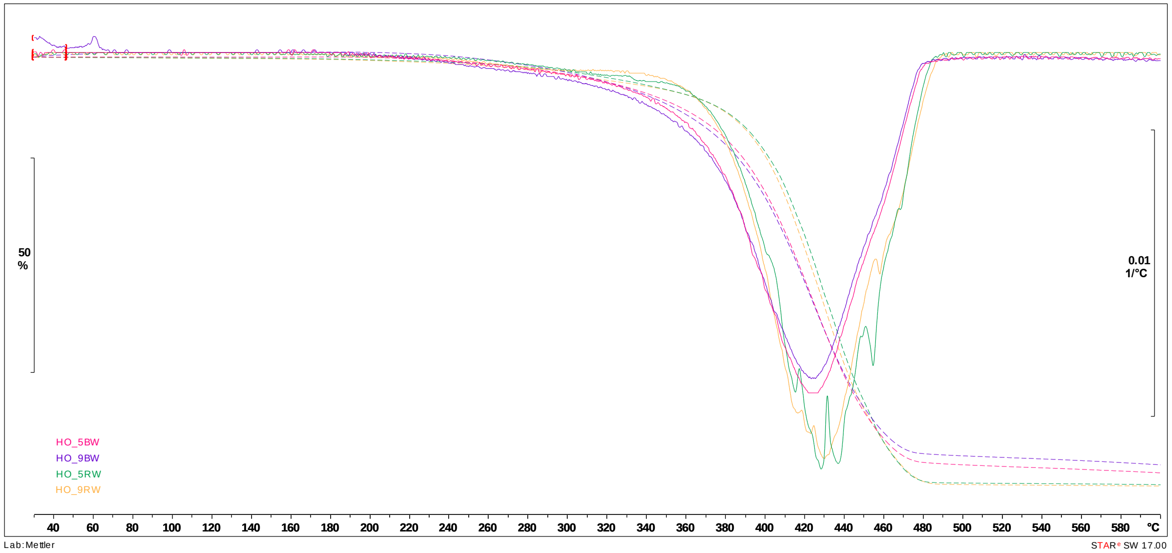

50
%
0.01
1/°C
HO_5BW
HO_9BW
HO_5RW
HO_9RW
40 60 80 100 120 140 160 180 200 220 240 260 280 300 320 340 360 380 400 420 440 460 480 500 520 540 560 580 °C
Lab:Mettler
STAR® SW 17.00

B

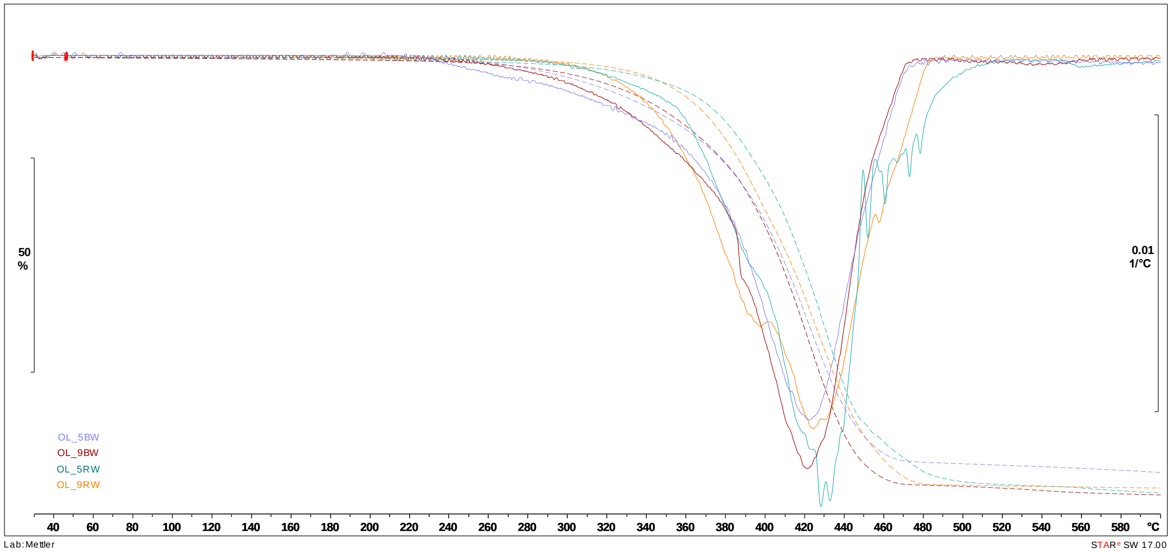

50
%
0.01
1/°C
OL_5BW
OL_9BW
OL_5RW
OL_9RW
40 60 80 100 120 140 160 180 200 220 240 260 280 300 320 340 360 380 400 420 440 460 480 500 520 540 560 580 °C
Lab: Mettler
STAR® SW 17.00

C

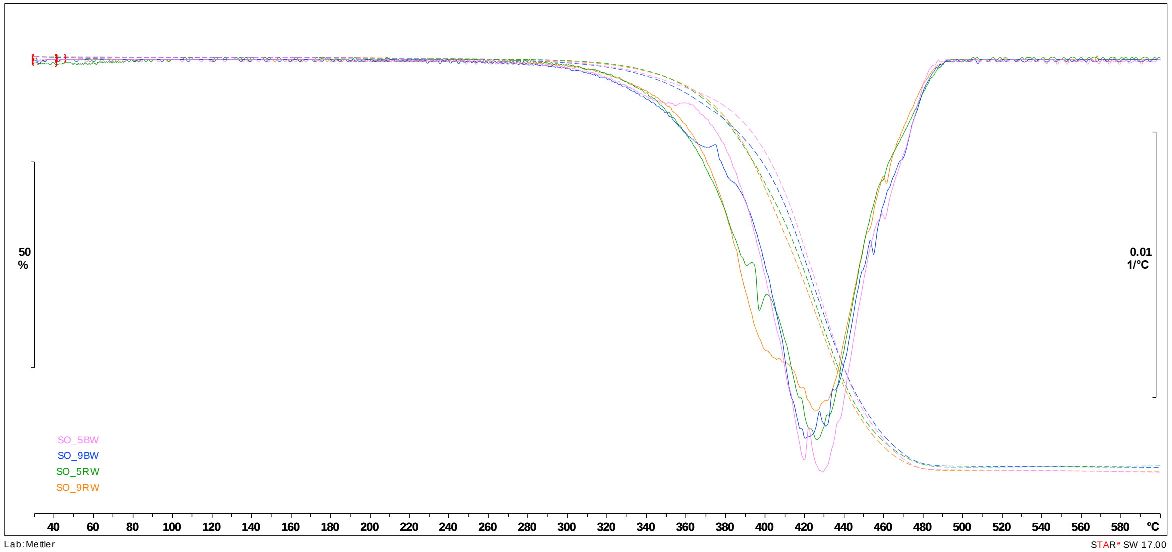

50
%
0.01
1/°C
SO_5BW
SO_9BW
SO_5RW
SO_9RW
40 60 80 100 120 140 160 180 200 220 240 260 280 300 320 340 360 380 400 420 440 460 480 500 520 540 560 580 °C
Lab: Mettler
STAR® SW 17.00

D

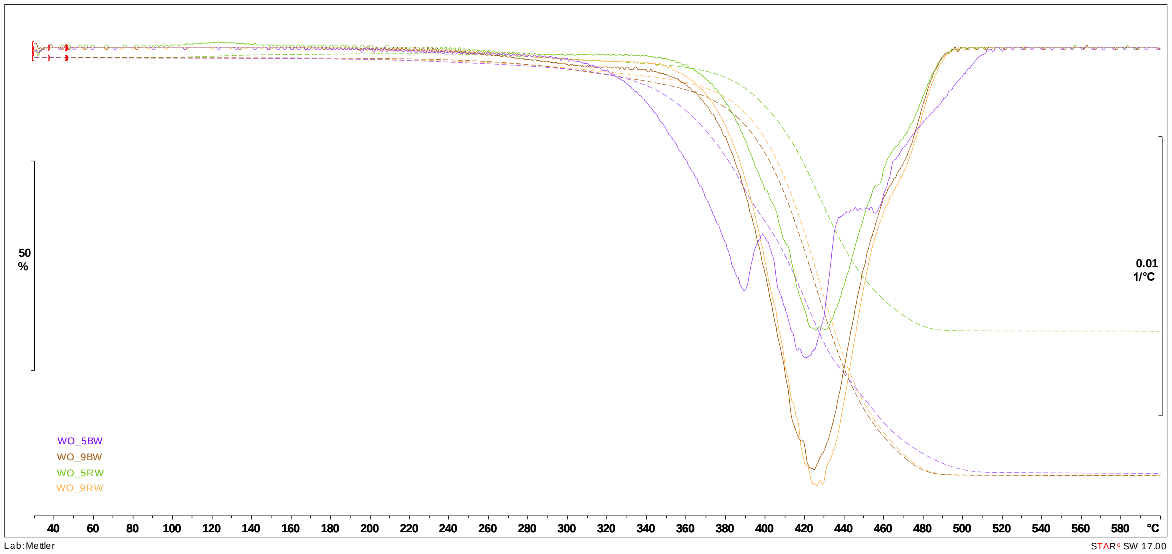

E

Figure S2. TGA (dashed line)/DTG (solid line) thermograms for oleogels formulated with different types of oil and wax
